# Hyperspectral Image Classification Using Deep Genome Graph-Based Approach

**DOI:** 10.3390/s21196467

**Published:** 2021-09-28

**Authors:** Haron Tinega, Enqing Chen, Long Ma, Richard M. Mariita, Divinah Nyasaka

**Affiliations:** 1 School of Information Engineering, Zhengzhou University, No. 100 Science Avenue, Zhengzhou 450001, China; tinegaharon@gmail.com (H.T.); ielongma@zzu.edu.cn (L.M.); 2 Henan Xintong Intelligent IOT Co., Ltd., No. 1-303 Intersection of Ruyun Road and Meihe Road, Zhengzhou 450007, China; 3 Microbial BioSolutions, 33 Greene Street, Troy, NY 12180, USA; richard.mariita@microbialbiosolutions.com; 4 The Kenya Forest Service, Nairobi P.O. Box 30513-00100, Kenya; dondieki@kenyaforestservice.org

**Keywords:** convolutional neural networks, hyperspectral images, hyperspectral image classification, spectral–spatial features, hybrid convolution networks, genome graphs

## Abstract

Recently developed hybrid models that stack 3D with 2D CNN in their structure have enjoyed high popularity due to their appealing performance in hyperspectral image classification tasks. On the other hand, biological genome graphs have demonstrated their effectiveness in enhancing the scalability and accuracy of genomic analysis. We propose an innovative deep genome graph-based network (GGBN) for hyperspectral image classification to tap the potential of hybrid models and genome graphs. The GGBN model utilizes 3D-CNN at the bottom layers and 2D-CNNs at the top layers to process spectral–spatial features vital to enhancing the scalability and accuracy of hyperspectral image classification. To verify the effectiveness of the GGBN model, we conducted classification experiments on Indian Pines (IP), University of Pavia (UP), and Salinas Scene (SA) datasets. Using only 5% of the labeled data for training over the SA, IP, and UP datasets, the classification accuracy of GGBN is 99.97%, 96.85%, and 99.74%, respectively, which is better than the compared state-of-the-art methods.

## 1. Introduction

Hyperspectral imaging is a combination of spectroscopy and imaging technologies. It involves using remote sensors to acquire a hyperspectral image (HSI) over the visible, near-infrared, and infrared wavelengths to specify the complete wavelength spectrum at each point on the earth’s surface [1]. Several efforts toward the enhancement of smart cameras/sensors have been made over the past decades to produce high-quality hyperspectral image data for Earth Observation (EO) [2]. The recent improvement in camera technology that utilizes complementary metal oxide semiconductor (CMOS) technology and multi-camera schemes has resulted in even more sophisticated smart sensors that use innovative algorithms such as adaptive cloud correction, which makes them adaptable to dynamic conditions with uncertain geometric changes and vibrations [3]. When the vision system or imaging device is combined with the main image processing unit, the resulting sensor is called the smart camera/sensor. These advancements have led to improvements in image resolution, acquisition speed, and the capability of providing images in which single pixels provide information from across the electromagnetic spectrum of the scene under observation, which in turn has improved the quality and speed of hyperspectral image processing [1]. The HSI is acquired by moving the vision system across the earth surface. The smart sensor raster-scans each scene in an image plane to extricate unique spectral signatures, using thousands of spectral bands recorded in different wavebands, creating a complete hyperspectral image data cube $I\in {ℛ}^{W\times H\times L}$, where (W×H) are the HSI pixels, and each $I_{i}\in \;{ℛ}^{L}$ records the spectral signature of the observed material.

Hyperspectral imaging technology has emerged as an effective tool for remote sensing applications, such as forestry, environmental monitoring [4], security [5], geology [6], ocean [7], precision agriculture [8], and many more. Unlike natural color images, hyperspectral images contain hundreds of channels that provide spectral information and detailed spatial cues [9]. The major benefit of hyperspectral images is that the unique spectral signatures obtained from certain objects enhances the detection of materials that make up a scanned object on the Earth’s surface, providing a much-improved comprehension of the scene under investigation. The process of analyzing the variegated land cover in hyperspectral images or data is called hyperspectral image classification (HSIC) [9]. An HSIC process begins with the image acquisition phase, followed by the feature extraction and learning phase. The extracted robust and invariant spectral–spatial features are then finally sent to the classifier for classification purposes. Each pixel in a raw remote sensed image is assigned a land cover class label or theme. 

Currently, the application of deep learning in the HSIC process has resulted in improved classification results. To tap the benefits of the readily available spectral and spatial information in hyperspectral images, researchers developed deep 3D-CNN models for feature learning. Although these models achieved better hyperspectral image classification accuracy than 2D-CNNs, they are computationally complex and frequently overfit. Recent developments in hyperspectral image classification have seen replacing 3D-CNNs with low-cost 2D-CNNs, creating hybrid models that are less computationally complex. Moreover, biological graph genomes are known to radically enhance the scalability, speed, and accuracy of genomic analyses [10]. 

Inspired by the work of Schatz et al. [11] and Rakocevic et al. [10] on graph genomes, and following the work of Roy et al. [12] that promotes the development of the 2D/3D CNN hybrid models, we propose an innovative genome graph-based bottom-heavy hybrid model called the deep hybrid genome graph-based network (GGBN) for hyperspectral image classification. This model is bottom-heavy because it utilizes 3D-CNN at the bottom layers to simultaneously process spectral–spatial features and 2D-CNNs at the top layers to process spatial features. The GGBN attains comparable results in terms of efficiency and accuracy with the state-of-the-art HSIC methods, such as SSRN and HybridSN. 

Our contributions are two-fold: (a) The development of a bottom-heavy hybrid model for hyperspectral image classification, which utilizes 2D and 3D CNNs in its structure to reduce the model complexity radically; and (b) unlike the HybridSN that also utilizes the 2D and 3D CNNs in its structure, the GGBN, in addition, utilizes the biological genome graph structure in its the network design. The resulting network structure contains multiple streams that independently extract spectral–spatial features, the residual layers that solve the degradation problem in the network, and the intermediate feature fusion to extract more abundant features. We attest that this is the first research that uses biological genome graphs in hyperspectral image classifications. However, in terms of computational efficiency, even though the test time of GGBN is better than HybridSN over IP and UP datasets, its training time performance is worse than HybridSN. Moreover, given more training data samples, the classification accuracy of SSRN and HybridSN becomes comparable with the proposed model. Therefore, our model is robust using small training sample data.

The rest of this paper is organized as follows. Section 2 discusses the related work. Section 3 discusses the proposed method. Section 4 discusses the materials and methods. Section 5 discusses the experimental results and analysis. Section 6 contains the conclusion of this article. 

## 2. Literature Review

This section will present insights concerning feature extraction and learning in the HSIC process and genome biology. 

### 2.1. Extraction and Learning in the HSIC Process

Early works on hyperspectral image analysis relied solely on spectral cues for HSIC, resulting in the development of feature extraction approaches, such as independent component analysis (ICA) [13], linear discriminant analysis (LDA) [14], and principal component analysis (PCA) [15,16]. In addition, this led to the development of pixel-wise classification methods, such as multinomial logistic regression [17], support vector machines (SVM) [18], random subspace [19], and one-dimensional neural networks [20]. However, these methods gave unsatisfactory classification results because they did not utilize spatial information. 

The advancements in remote sensors have resulted in a drastic increase in research that considers spatial context information, which can significantly increase HSIC accuracy. The strategies for the extraction of spatial context information can be classified into handcrafted or deep learning. Most handcrafted spatial context feature extraction can be classified under neighborhood window [21], Markov random field (MRF) [22], segmentation [23], morphological, and texture features [24]. However, the handcrafted spatial feature extraction methods lack the discriminative power available in deep learning features for the problem of HSIC. 

The application of deep learning in the field of machine learning and pattern recognition has achieved tremendous results, especially in tasks such as object detection [25], image analysis [26], and natural language processing [27], promoting their development in hyperspectral remote sensing tasks. In hyperspectral remote sensing, deep learning approaches are introduced into the HSIC problem to learn hierarchical representations [28]. Recent research in deep remote sensing tasks has considered the spectral and spatial information available in HSI for classification purposes. Several researchers, such as Chen et al. [29], Li et al. [30], and Hamida et al. [31], among many others, have proposed the use of deep 3D-CNN-based approaches to extract spectral–spatial feature maps for HSIC. Although they achieved a state-of-the-art result compared with the 2D-CNN-based methods, 3D-CNN-based approaches are complex and computationally expensive in parameter usage and speed. Moreover, since most of the existing 3D CNN-based approaches have stacked 3D-CNNs in their structure, they cannot optimize the estimation loss directly through such a nonlinear structure [28]. This resulted in the development of hybrid models that combined 2D-CNNs with 3D-CNNs in their structure. 

Several hybrid-based approaches take advantage of both the 2D and 3D CNN to achieve better accuracy in HSI analysis. For instance, Roy et al. [12] proposed a HybridSN model that uses the 3D-CNN layers at the bottom layers of the network to simultaneously process spectral–spatial features and 2D-CNN layers at the top of the architecture to process the spatial features. Yang et al. [28] combined 2D and 3D CNNs in the model structure to develop a hybrid 2D/3D CNN. 

### 2.2. The Biological Genome Graphs

Genomic tools have enabled the elucidation of the properties and distribution of common and rare genetic variations. The insights provided help to explain genetic diversity and empower humanity to understand disease biology [32]. This is made possible by algorithms that can enable the building of variant-aware graph genomes [33]. The implementation of genome graphs obtained after the alignment of sequence reads has enabled genomic experts to map and decipher structural variations in genomes [10]. Generally, a genome graph is a directed sequence graph used in genomic analyses [10]. Genome sequencing is a term that combines two words: genome, which refers to all of the DNA molecules in an organism’s cells, and sequencing, which refers to the scientific process of identifying the sequence composition of biomolecules, including RNA, protein, and DNA. Pertaining to genome assembly, genome sequencing is a computational process that generally follows a hierarchical approach of entirely or nearly entirely deciphering the DNA sequence of an organism’s genome at a single time using numerous short sequences called reads derived from the portions of the target DNA as input. The advancements have accelerated this process in new technologies that provide an extensive view of the gene space of organisms. 

In plants, the first experiment in sequencing was done using first-generation automated DNA sequencing instruments on thale cress [34], maize [35], rice [36], and papaya [37]. Unlike the first-generation sequencing instruments, the second-generation sequencing instruments, which are the current state-of-the-art, sequence billions of bases per day for millions or billions of dollars per gigabase [38]. These sequencing instruments have been utilized to study the volumes of plant genomes, allowing for rich gene network annotation [39], plant breeding optimization [40], and use in research that utilizes genome sequences as the basis of analyses [41].

Assembling a voluminous genome, especially in plants, is operationally complicated due to enormous error correction and filtering demands, considerable computational resources, and susceptibility to the parameters used. Moreover, plants genomes are innately sophisticated due to their high diversity [42] and higher rates of heterozygosity and ploidy, which are absent in other kingdoms [11]. To overcome these challenges, several techniques that implicitly or explicitly borrow ideas from graph-based models, which we collectively refer to as genome graphs, have been devised to represent and organize data gleaned from these cohorts. A genome graph is constructed from a population of genome sequences, such that a sequence path represents each haploid genome in this population through the graph [43]. Genome graphs use graph alignment, which can correctly position all reads on the genome, as opposed to linear alignment, which is reference-based and cannot align all reads or use all the available genome data. Graph genomes can improve the volume of aligned reads, resolve haplotypes, and create a more accurate depiction of population diversity [43]. Rakocevic et al. [10] experimentally demonstrated that graph genome references improve read mapping accuracy, as well as increase variant calling recall without any loss in precision. Therefore, it is clear that graph genomes, if used appropriately, can radically enhance the scalability and accuracy of genomic analyses. Genome graphs improve the representation of assembled genomes in plant genome sequencing by providing graph-centric and population-aware formats that can express the intricacies of plant genomes, especially the partially assembled ones [44,45].

Incorporating the genome approach into hyperspectral image classification can improve classification results. It is from this perspective that we sought to experimentally investigate the contribution of genome graphs and hybrid 2D/3D CNN in the feature learning of hyperspectral remote sensing images. We propose a deep hybrid genome graph-based network (GGBN) for hyperspectral image classification. The GGBN attains comparable results in terms of efficiency and accuracy with the state-of-the-art HSIC methods, such as SSRN and HybridSN. 

## 3. The Proposed Model Framework

The proposed (GGBN) model is divided into preprocessing, feature extraction, and classification sections.

### 3.1. The Preprocessing Section

The preprocessing section involves the dimensionality reduction of the bands using the principal component analysis (PCA) and neighborhood extraction to extract overlapping 3D patches.

**Figure 1:**
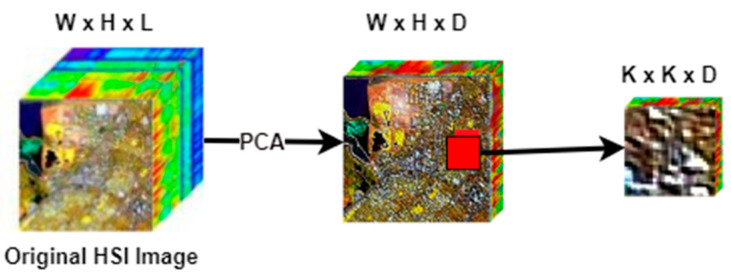
Preprocessing part.

As shown in Figure 1, the preprocessing section involves the dimensionality reduction of the bands using the principal component analysis (PCA). Once the depth of the HSI data cube is reduced, then we extract overlapping 3D patches.

Let the original HSI data cube be denoted as $I\in {ℛ}^{W\times H\times L}$, where *W* is the width, *H* is the height, and *L* is the number of spectral bands. Every HSI pixel in I is made up of *L* spectral bands, which form a one-hot label vector $\;Z=\left(z1,z2,\ldots \mathrm{zC}\right)\;\in {\;R}^{1\times 1\times C}$, where C is the class categories for each dataset. Image cube I contains high spectral redundancy due to high levels of interclass similarity and intraclass variability. 

To reduce the spectral dimension’s redundancy, we apply PCA to the original HSI data cube I, resulting in data cube B with dimensions  W×H×D, where  D<L. Before we apply PCA, we begin by transforming the original HSI data cube I dimensions into a two-dimensional matrix, M×L where *M* is the number of pixels obtained by multiplying  W×H, and L remains to represent the number of spectral bands. The first step in PCA involves centering and standardizing the original hyperspectral image data by demeaning. This is achieved by computing and eliminating the average value of every spectral band in the original data cube (see line 2 of Algorithm 1). The next step involves computing the covariance matrix, which is the product of the preprocessed data matrix and its transpose (see line 3). This step is immediately followed by the extraction of eigenvectors associated to the covariance matrix (see line 4). Finally, the dimensionality reduction is achieved by projecting every single pixel of the original hyperspectral image data cube onto a subset of eigenvectors (see lines 5 and 6).
**Algorithm 1: Principle Component Analysis (PCA)****1****Input:** Original Hyperspectral image  I(M×L), *M* pixels, *L* spectral bands.**2**Centre and standardize  I, putting it into matrix  V.**3**Compute the covariance matrix $C=\frac{1}{L}V^{T}V$
**4**Compute the eigenvalues and eigenvector of *C*, such that $E=Y^{-1\;}CY$, where Y holds the eigenvectors of  C, and E is the M×M diagonal eigenvalue matrix.**5**Sort *D* into the order of decreasing eigenvalues, and apply the same order to *V*.**6**Eigenvalues less than some η are rejected, leaving *D* dimensions in data which is the new dimensional feature subspace.**7****Output:** Reduced Hyperspectral image B(M×D), where D<L and $M\in {ℛ}^{W\times H}$.

The new data cube $B\in {ℛ}^{W\times H\times D}$ is further divided into G small overlapping patches of spatial dimension K×K and depth D, of which the label of the central pixel decides the truth labels at the spatial location (x,y). 

### 3.2. Genome Graph-Based Network (GGBN) 

**Figure 2:**
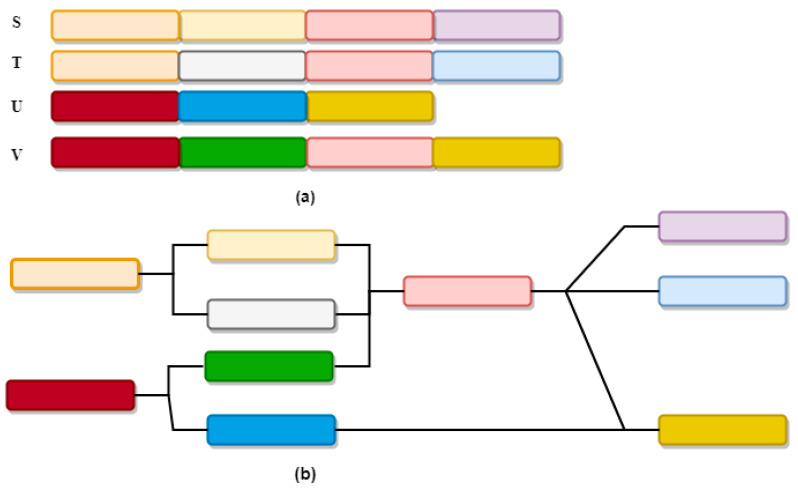
(**a**) tetraploid genome (**b**) assembly graph.

According to Schatz et al. [11], a tetraploid genome with homozygosity/heterozygosity shown as variegated blocks (see Figure 2a) can be intertwined to form a complex pattern of the assembly graph without repeats or sequencing error (see Figure 2b). 

**Figure 3:**
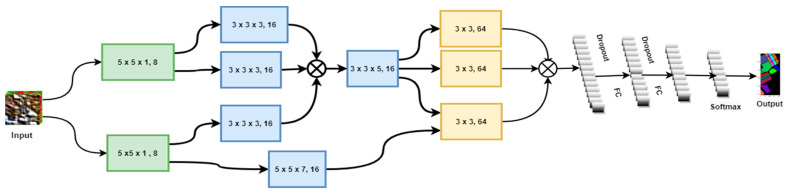
The architectural design of the deep genome graph-based network (GGBN) for hyperspectral image classification.

The design of the genome graph-based network (GGBN) (see Figure 3) that efficiently extracts highly discriminative HSI features, then flattens the output before passing it to fully connected layers to learn deep features, and later to the softmax layer for classification was inspired by the research work of Schatz et al. [11].

The input to the network is a 3D patch of size  K×K×D. Here, K is the length and the width and D is the depth of the input patch. The first layer extracts spatial features using a 3D filter in the proposed network, while the rest of the remaining layers extract spectral–spatial features using 3D kernels and later 2D kernels, as illustrated in Figure 3 above. GGBN uses a residual layer between layer two and layer three to recover lost features at the third convolution layer (see Figure 3). In addition, the model structure implements a feature fusion at different network points that results in better classification accuracy. The output from the fourth layer is flattened before being passed to fully connected layers and later to the softmax layer for feature learning and classification, respectively. The output from each layer is passed through an activation function to introduce nonlinearity. The activation value at spectral–spatial position (x,y,z) in the $j^{\mathrm{th}}$ feature map of the $i^{\mathrm{th}}$ layer denoted as $v_{i,j}^{x,y,z}$, is given by:

(1) $${\;v}_{i,j}^{x,y,z}=\;R\left(b_{i,j}+\overset{M}{\underset{m=1}{\sum }}\overset{P_{i}-1}{\underset{p=0}{\sum }}\overset{Q_{i}-1}{\underset{q=0}{\sum }}\overset{R_{i}-1}{\underset{r=0}{\sum }}w_{i,j,m}^{p,q,t}\times v_{\left(i-1\right),m}^{\left(x+p\right),\left(\;y+q\right),\left(z+r\right)}\right)$$

where parameters $P_{i},{\;Q}_{i},{\;\mathrm{and}\;R}_{i}$ are the width, the height, and the depth of the kernel, respectively. Parameter $b_{i,j}$ is the bias value for the $j^{\mathrm{th}}$ feature map of the $i^{\mathrm{th}}$ layer and M is the total number of feature maps in the ${\left(l-1\right)}^{\mathrm{th}}$ layer connected to the current feature map. $w_{i,j,m}^{p,q,r}$ is the value of the weight parameter for position (p,q,r) kernel connected to the $m^{\mathrm{th}}$ feature map in the previous layer. 

To introduce nonlinearity in the 2D layer, the convolved feature maps are passed through the ReLU activation function such that the activation value at position (x,y) in the $j^{\mathrm{th}}$ spatial feature map of the $i^{\mathrm{th}}$ CNN layer is symbolized as $v_{i,j}^{x,y}$ and can be generated using the equation:

(2) $$v_{i,j}^{x,y}=\;R\left(b_{i,j}+\overset{M}{\underset{m=1}{\sum }}\overset{P_{i}-1}{\underset{p=0}{\sum }}\overset{Q_{i}-1}{\underset{q=0}{\sum }}w_{i,j,m}^{p,q}\times v_{\left(i-1\right),m}^{\left(x+p\right),\left(\;y+q\right)}\right)$$

where R is the ReLU activation function. The value $w_{i,j,m}^{\mathrm{pq}}$ is the weight parameter for spatial position (p,q) kernel connected to the previous layer’’s $m^{\mathrm{th}}$ feature map. 

## 4. Materials and Methods

In this section, we present the detailed configuration description of the three publicly available HSI datasets, namely Indian Pines (IP), University of Pavia (UP), and Salinas (SA), used in this research. We use the overall accuracy (OA), average accuracy (AA), and the kappa coefficient (*k*) to evaluate the performance of the models across the three datasets. OA gives the percentage of the correctly classified samples, AA is per class accuracy presented in percentage, and *k* involves commission and omission errors and illustrates the classifier’s overall performance. For all three evaluation metrics, a higher value represents better accuracy.

### 4.1. Description

We train and test the performance of the proposed method and competing state-of-the-art methods on the three publicly available HSI datasets: Indian Pines (IP), University of Pavia (UP), and Salinas (SA). 

The Indian Pines (IP) dataset was collected by the Airborne Visible Infrared Imaging Spectrometer (AVIRIS) sensor with a spatial resolution of 20 meters flying over the Indian Pines site area Northwestern Indiana. It has a spatial dimension of 145×145 pixels with 224 spectral bands ranging from 0.4 to 2.5 μm. After eliminating 24 spectral bands covering the water absorption region, the resulting hyperspectral data cube dimension is 145×145×200. Its ground truth data contain 16 classes of vegetation. 

The University of Pavia (UP) dataset was collected by reflective optics system imaging spectrometer-03 (ROSIS-03) sensors with a spatial resolution of 1.3 meters flying over the University of Pavia. The resulting hyperspectral data contain 135 spectral bands collected in a wavelength range of 0.43–0.86 μm and a spatial resolution of 610×340 pixels. Once 12 water absorption bands are discarded, the hyperspectral data cube’s resulting dimension is  610×340×103. The University of Pavia scene consists of 9 classes, with almost all classes having more than 1000 labeled pixels. The AVIRIS sensor with a 3.7 spatial resolution acquired the Salinas Scene (SA) dataset over Salinas Valley, CA, USA. The SA dataset contains 224 spectral bands and 512×217 pixels spatial dimension. The spectral bands’ wavelengths range from 0.36 to 2.5 μm. Once 20 water-absorbing spectral bands are discarded, the resulting hyperspectral data cube dimensionality is  512×217×204. The ground truth data include a total of 16 classes.

### 4.2. Parameter Settings

All experiments are conducted online using Google Colab Inc. We randomly divide sample data into training and testing for all the three experimental datasets, namely IP, UP, and SA. We compare classification results of the proposed method with the state-of-the-art methods on 5% training and 95% testing data. We selected the optimal parameters based on the classification outcome. We chose the Adam optimizer method with a learning rate of 0.0005 for UP and 0.001 for both SA and IP. We used batch sizes 64, 256, and 256 to train the network for 100, 150, and 150 epochs on IP, SA, and UP datasets. Finally, the dropout is set to 0.6 for IP and SA and 0.8 for UP.

## 5. Experimental Results and Discussion

This section reports the quantitative and qualitative results of the proposed GGBN and a comparison with the other state-of-the-art methods on IP, UP, and SA datasets. We will compare the performance of the proposed model with state-of-the-art methods, such as SSRN [46] and HybridSN [12]. We selected these two models because the classification performance of SSRN and HybridSN is far higher than that of previously studied methods, such as 2D-CNN, 3D CNN [31], and M3D-DCNN [47] models

**Figure 4:**
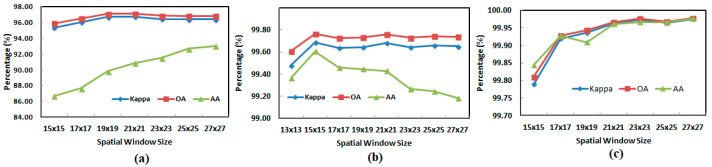
The effect of the spatial window size on the performance (in percentage) of the GGBN model over the (**a**) IP, (**b**) UP, and (**c**) SA datasets.

Figure 4 provides the performance summary (in percentage) when varying the spatial dimensions of the overlapping 3D patch of the GGBN model over IP, UP, and SA datasets on 5% training and 95% testing sample data. 

From Figure 4, we observed that considering the OA, AA, and Kappa, the optimal performance of the GGBN over IP, UP, and SA datasets is achieved when the spatial dimensions of the overlapping 3D patches of the input volume are set to 23 × 23 × 30, 15 × 15 × 15, and 23 × 23 × 15 respectively.

Table 1 summarizes the training and testing time in seconds of SSRN, HybridSN, and the proposed GGBN models over the IP, UP, and SA datasets on 5% training and 95% testing sample data.

The computational efficiency in terms of training and testing time (in seconds) shown in Table 1 shows that the proposed method in terms of training time performance is better than SSRN and worse than HybridSN. The proposed model performance in test time is better than HybridSN over IP and UP datasets. Therefore, we can conclude that the computational efficiency of the proposed model is comparable with that of SSRN and HybridSN. 

**Figure 5:**
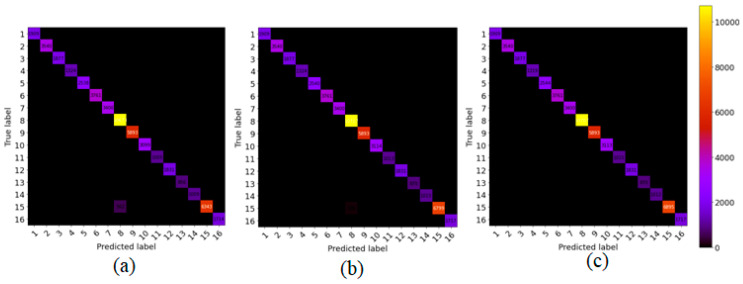
The confusion matrix for classification results of (**a**) SSRN, (**b**) HybridSN (**c**) GGBN over IP dataset.

**Figure 6:**
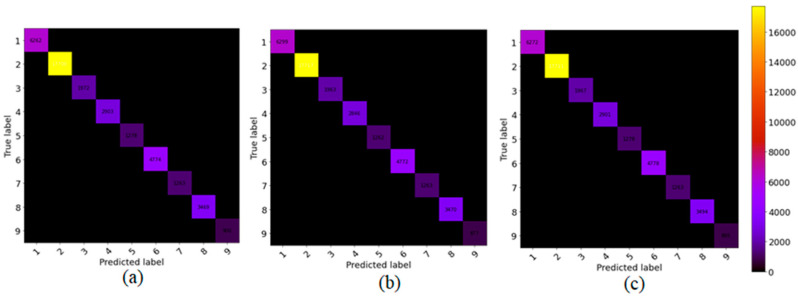
The confusion matrix for classification results of (**a**) SSRN, (**b**) HybridSN (**c**) GGBN over UP dataset.

**Figure 7:**
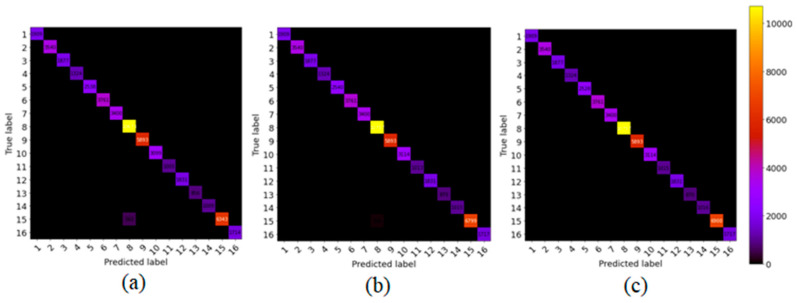
The confusion matrix for classification results of (**a**) SSRN, (**b**) HybridSN (**c**) GGBN over SA dataset.

To show the robustness of the proposed method, we compare the proposed model with the other state-of-art methods, such as SSRN and HybridSN, on 5% training sample data and test on the remaining (95%) portion. Figure 5, Figure 6 and Figure 7 show the robustness of the proposed (GGBN) model in feature learning even with low (5%) training sample data. 

We observe in Figure 5, Figure 6 and Figure 7 that most of the sample data lie in the diagonal line even with low training data. Therefore, the majority of the sample data were correctly classified. This demonstrates the robustness of the proposed model over small training sample data.

**Figure 8:**
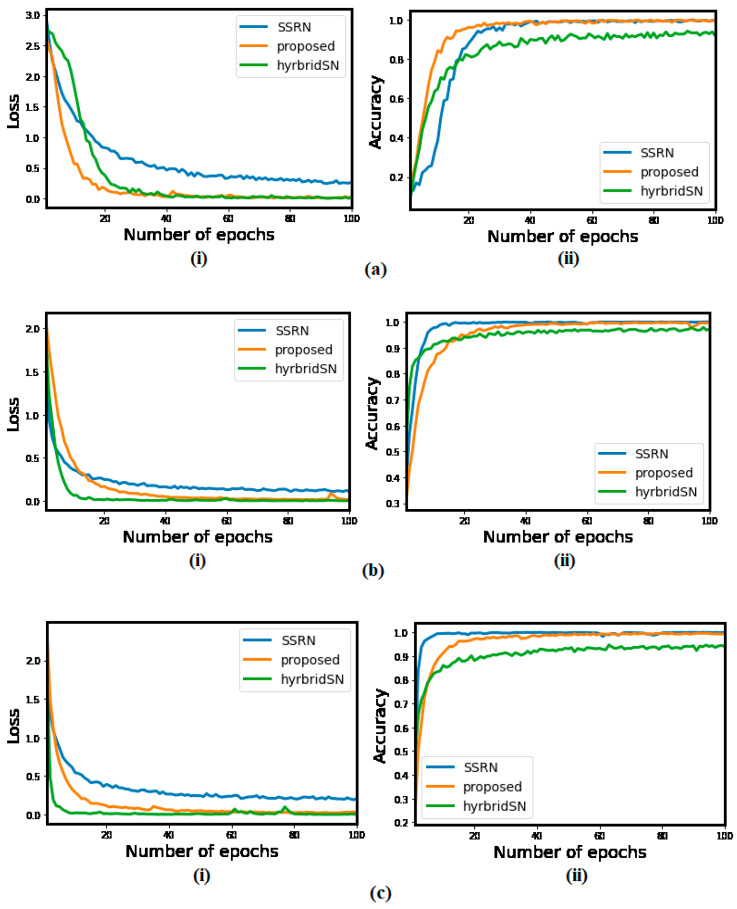
The loss and accuracy convergence graphs of the SSRN, Hybrid, and GGBN for each epoch over the (**a**) IP, (**b**) UP, and (**c**) SA datasets.

Further, the accuracy and loss convergence graphs shown in Figure 8 illustrate that the GGBN converges faster than SSRN and HybridSN using the IP dataset, and second with the UP and SA datasets. 

### 5.1. Classification Results for the Indian Pines (IP) Dataset

Table 2 shows the class-specific classification accuracies of M3D-CNN, SSRN, HybridSN, and GGBN using the IP image. The representative classification maps are provided in Figure 10.

It can be observed in Table 2 that the proposed method outperforms M3D-CNN, SSRN, and HybridSN in terms of OA, AA, and Kappa. The GGBN improves the OA, AA, and Kappa of HybridSN by 3.13%, 3.89%, and 3.59%, respectively. In comparison, the SSRN is improved by 4.2%, 3.5%, and 16.12%, respectively. The OA, AA, and Kappa of M3D-CNN are improved by the most significant margin of 29.43%, 20.76%, and 18.94%, respectively. For similar classes, such as Grass-tress, Grass-pasture, and Grass-pasture mowed, the proposed (GGBN) model records a higher performance of 5.14%, 0.39%, and 14.8%, respectively, than those obtained by the HybridSN method. Similar performance trends can be observed over Soybeans-no till, Soybeans-min till, and Soybeans-clean till classes. The result demonstrates the superiority of our model structure on datasets characterized by small samples and classes with similar textures across multiple bands.

**Figure 9:**
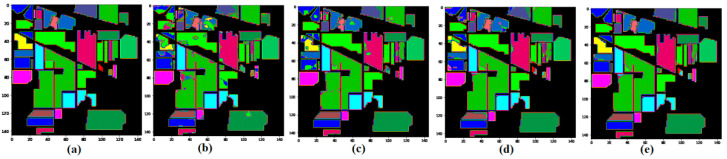
Classification maps of Indian Pines data set: (**a**) Ground truth; (**b**) M3D-CNN; (**c**) SSRN; (**d**) HybridSN; (**e**) GGBN.

We observe in Figure 9 that the M3D-CNN, SSRN and HybridSN have more noisy, scattered points in the classification maps, unlike the GGBN methods. Therefore, the proposed method can remove the noisy scattered points and lead to smoother classification results without blurring the boundaries.

### 5.2. Classification Results for the University of Pavia (UP) Dataset

**Figure 10:**
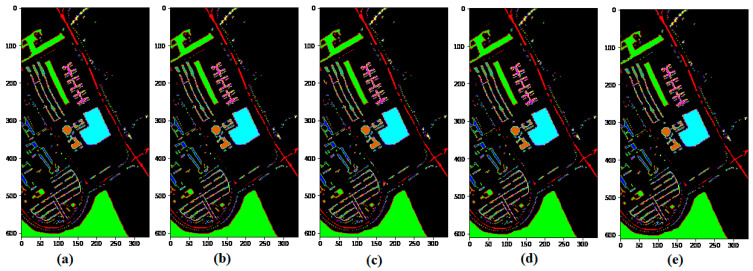
Classification maps of the University of Pavia (UP) dataset: (**a**) Ground truth; (**b**) M3D-CNN; (**c**) SSRN; (**d**) HybridSN; (**e**) GGBN.

Table 3 provides a summary of the classification results of M3D-CNN, SSRN, HybridSN, and GGBN models with 5% training and 95% testing over the UP dataset. The classification map accuracies are illustrated in Figure 10. 

**Figure 11:**
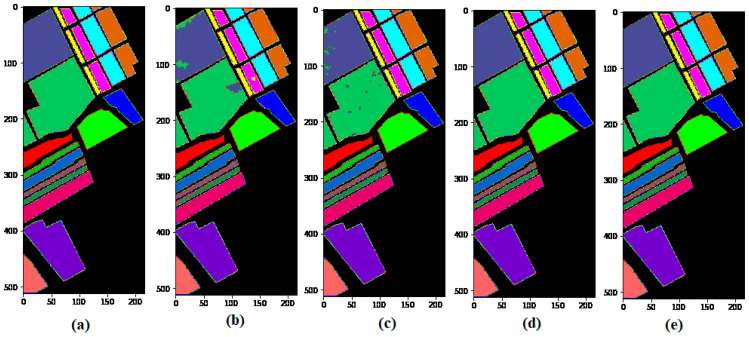
Classification maps of the Salinas (SA) dataset: (**a**) Ground truth; (**b**) M3D-CNN; (**c**) SSRN; (**d**) HybridSN; (**e**) GGBN.

It can be seen in Table 3 that our proposed method attains the best classification accuracy as compared to M3D-CNN, SSRN, and HybridSN methods with 5% training sample data. Moreover, we observe in Figure 10 that the compared methods produced almost identical classification maps to the ground truth at 5% training sample data. Table 3 and Figure 11 demonstrate the robustness of the proposed method over the UP dataset.

### 5.3. Classification Results for the Salina Scene (SA) Dataset

Table 4 shows the classification results obtained by different classifiers for the SA datasets, and the resultant maps are provided in Figure 11. 

It can be observed in Table 4 that under the condition of the same training samples, the proposed method records the highest results compared with the M3D-CNN, SSRN, and HybridSN in terms of OA, AA, and Kappa. The better performance of the GGBN model proves the capacity and effectiveness for multiple feature learning.

From Figure 11, we observe that, unlike the M3D-CNN, and SSRN that introduces some “salt and pepper” to the classification map, the HybridSN and GGBN model produces an almost identical classification map to the ground truth with 5% training sample data over the SA dataset. This demonstrates the ability of the proposed (GGBN) model to correctly classify the majority of the class labels using small training sample data. 

### 5.4. Model Performance on Varied Training Sample Data over IP, UP, and SA Datasets

To further demonstrate the robustness of the proposed method, we randomly select 1%, 3%, 5%, 10%, and 20% of the data for training and test on the remaining portion for SA and UP. However, for the IP dataset we omit 1% training sample data. The resulting performance is as shown in Table 5, Table 6 and Table 7. 

Table 5, Table 6 and Table 7 show that M3D-CNN has the lowest classification accuracy compared to all the other models, which can be attributed to its network structure nature that utilizes only multi-scale 3D-CNN layers. The SSRN method performs better than M3D-CNN because it uses residual connections to extract deep spatial and spectral features. The effectiveness of combining the 2D and 3D convolutional layers is evidenced by the higher classification accuracy attained by the HybridSN model. The GGBN method attains better classification accuracy than all the other models across all the experimental datasets. We can attribute the performance to the genomic structural network design that combines the benefit of residual network layers, a more comprehensive structural network, intermediary feature fusion, and the use of both 2D and 3D convolutional layers. We also observe that the classification accuracy of all compared models decreased with the training sample proportion. However, the decreasing speed varies across different models. For instance, with 5% training sample in IP, GGBN improves OA of HybridSN by 3.13%, and as the training sample amounts are decreased, the margin of accuracy improvement becomes even more pronounced. We note the improvement of IP accuracy from 3.13% to 6.43% with 5% and 3% training samples, respectively. The same trend is observed on PU and SA datasets.

**Figure 12:**
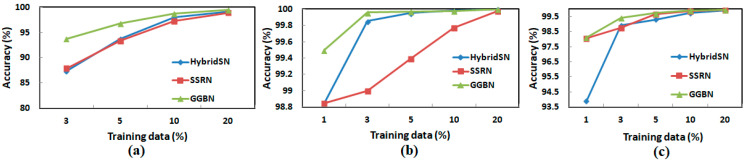
Overall accuracy (OA) in percentage under different numbers of training data (**a**) IP, (**b**), SA, and (**c**) UP.

Further, Figure 12 graphically shows the accuracy behavior under different training sample portions. We note GGBN models fall at the slowest speed, which shows the robustness of the model in hyperspectral image classification. 

## 6. Conclusions

This research has proposed an innovative deep genome graph-based network (GGBN) for hyperspectral image classification. The GGBN contains three sections, namely (a) the preprocessing section that involves the dimensionality reduction of the bands using the principal component analysis (PCA), and later the extraction of the overlapping 3D patches that are input into the model structure; (b) the feature learning section that is inspired by the performance of the genome graphs in radically enhancing the scalability and accuracy of genomic analyses, and the achievements of hybrid 2D/3D CNN in feature learning of hyperspectral remote sensing images; and (c) the classification section that uses the softmax function. 

The GGBN uses the biological genome graph-based structure in its network to extract spectral–spatial features of hyperspectral images, resulting in increased classification performance over the IP, UP, and SA datasets compared with the state-of-the-art methods such as M3D-CNN, SSRN, and HybridSN. We observed that the proposed GGBN method performed even better with insufficient training sample data than other state-of-the-art methods (i.e., M3D-CNN, SSRN, and HybridSN), which confirms the superiority of the GGBN method with extensive and minimal training data. Moreover, the GGBN outperformed the M3D-CNN, SSRN, and HybridSN in classifying similar classes, unlike the SSRN and HybridSN, which introduce some “salt and pepper” to the classification map when the training data are small. The proposed model produces an almost identical classification map to the ground truth. This shows that GGBN has higher model representation ability than the M3D-CNN, SSRN, and HybridSN models. The strength of the GGBN model lies in its structural nature that allows multiple streams that independently extract spectral–spectral features, the residual layer that solves the degradation problem in the network, and intermediate feature fusion to extract more abundant features. We attest that this is the first research that uses the biological genome graphs in hyperspectral image classification. However, in terms of computational efficiency, the GGBN lags behind the HybridSN, even though its test time is better than HybridSN over IP and UP datasets. Therefore, more research needs to be conducted on the use of various biological genome graphs to enhance the structure of hyperspectral classifiers and prove their credibility. In the near future, we will make an effort to run the model using various hyperspectral datasets and compare it with other state-of-the-art methods to prove its robustness.

## Figures and Tables

**Table 1:** The training and testing time in seconds over IP, UP, and SA datasets using SSRN, HybridSN, and GGBN model.

Data	SSRN		HybridSN		GGBN	
	Train	Test	Train	Test	Train	Test
IP	70.9	1.5	36.9	2.7	163.5	2.5
UP	417.7	4.0	37.5	4.7	62.8	4.3
SA	527.7	4.9	45.4	5.6	227.8	7.4

**Table 2:** Classification Results (%) over IP dataset.

Class No.	Class Labels	Samples (Pixels)	Cover (%)	M3D-CNN (%)	SSRN (%)	HybridSN (%)	GGBN (%)
1	Alfalfa	46	0.45	30.75	12.73	55.23	46.36
2	Corn-no till	1428	13.93	72.76	92.73	92.97	94.78
3	Corn-min till	830	8.1	61.76	93.59	90.18	98.38
4	Corn	237	2.31	57.46	72.80	84.22	94.49
5	Grass-pasture	483	4.71	85.19	98.19	94.01	99.15
6	Grass-trees	730	7.12	92.13	99.67	97.63	98.02
7	Grass-pasture-mowed	28	0.27	45.54	0.74	73.70	87.78
8	Hay-windrowed	478	4.66	94.01	99.82	99.76	99.85
9	Oats	20	0.2	20.45	0.00	88.95	78.95
10	Soybean-no till	972	9.48	70.97	91.54	95.41	97.82
11	Soybean-min till	2455	23.95	76.75	95.21	97.08	97.98
12	Soybean-clean	593	5.79	59.35	87.83	82.02	92.97
13	Wheat	205	2	94.36	98.62	94.31	97.64
14	Woods	1265	12.34	94.55	99.84	98.96	99.03
15	Buildings-Grass-Trees-Drives	386	3.77	51.21	83.68	79.84	92.29
16	Stone-Steel-Towers	93	0.91	64.39	81.25	77.61	88.75
	Kappa			66.98	92.39	92.82	96.41
	OA			76.09	93.35	93.72	96.85
	AA			72.57	75.39	87.62	91.51

**Table 3:** Classification Results (%) of UP Dataset.

Class No.	Class Labels	Samples (Pixels)	Cover (%)	M3D-CNN (%)	SSRN (%)	HybridSN (%)	GGBN (%)
1	Asphalt	6631	15.5	95.44	99.65	99.55	99.58
2	Meadows	18,649	43.6	93.98	99.96	99.98	99.95
3	Gravel	2099	4.91	90.36	98.14	98.69	98.41
4	Trees	3064	7.16	97.37	99.67	98.20	99.43
5	Painted_metal_sheets	1345	3.14	99.75	100	98.85	99.95
6	Bare_Soil	5029	11.76	92.14	100	99.99	99.99
7	Bitumen	1330	3.11	92.62	99.59	99.89	99.99
8	Self-Blocking_Bricks	3682	8.61	94.62	98.56	97.42	99.47
9	Shadows	947	2.21	99.24	99.97	94.21	99.57
	Kappa			95.06	99.57	99.10	99.65
	OA			92.50	99.68	99.32	99.74
	AA			90.19	99.50	98.46	99.59

**Table 4:** Classification Results (%) over SA dataset.

Class No.	Class Labels	Samples (Pixels)	Cover (%)	M3D-CNN (%)	SSRN (%)	HybridSN (%)	GGBN (%)
1	Brocoli_green_weeds	2009	3.71	97.35	100.00	100.00	100.00
2	Brocoli_green_weeds	3726	6.88	99.81	99.99	100.00	100.00
3	Fallow	1976	3.65	97.98	100.00	100.00	100.00
4	Fallow_rough_plow	1394	2.58	99.21	99.85	99.95	99.99
5	Fallow_smooth	2678	4.95	99.18	99.76	99.71	99.54
6	Stubble	3959	7.31	99.17	100.00	99.97	99.98
7	Celery	3579	6.61	99.21	99.99	99.99	99.99
8	Grapes_untrained_	11,271	20.82	87.9	99.29	99.99	100.00
9	Soil_vinyard_develop	6203	11.46	99.29	100.00	100.00	100.00
10	Corn_senesced_green_weeds	3278	6.06	94.13	99.82	99.98	100.00
11	Lettuce_romaine_4wk	1068	1.97	95.94	99.86	99.91	100.00
12	Lettuce_romaine_5wk	1927	3.56	98.11	100.00	100.00	100.00
13	Lettuce_romaine_6wk	916	1.69	97.4	99.92	99.87	99.99
14	Lettuce_romaine_7wk	1070	1.98	96.43	99.55	99.82	99.98
15	Vinyard_untrained	7268	13.43	79.97	96.99	99.87	99.96
16	Vinyard_vertical_trellis	1807	3.34	90.61	99.55	100.00	100.00
	Kappa			95.73	99.32	99.95	99.96
	OA			92.49	99.39	99.95	99.97
	AA			91.66	99.66	99.94	99.96

**Table 5:** Performance of the selected models on varied training sample data over IP dataset.

Model	Training Sample Data in Percentage			
	3%	5%	10%	20%
M3D-CNN	66.23	76.09	84.38	91.95
SSRN	87.89	93.35	97.30	98.93
HybridSN	87.35	93.72	97.97	99.16
GGBN	93.78	96.85	98.80	99.45

**Table 6:** Performance of the selected models on varied training sample data over UP dataset.

Model	Training Sample Data in Percentage				
	1%	3%	5%	10%	20%
M3D-CNN	86.29	90.78	92.50	93.82	94.60
SSRN	98.07	98.78	99.68	99.88	99.96
HybridSN	93.88	98.92	99.32	99.73	99.92
GGBN	98.13	99.42	99.74	99.92	99.95

**Table 7:** Performance of the selected models on varied training sample data over SA dataset.

Model	Training Sample Data in Percentage				
	1%	3%	5%	10%	20%
M3D-CNN	86.37	90.38	92.49	93.44	94.47
SSRN	98.85	99.00	99.39	99.77	99.97
HybridSN	98.85	99.85	99.95	99.98	100.00
GGBN	99.50	99.96	99.97	99.98	100.00

## Data Availability

All datasets used in this research are open accessible online (http://www.ehu.eus/ccwintco/index.php?title=Hyperspectral_Remote_Sensing_Scenes) (accessed on 22 September 2021).
